# Neutron Radiation Dose Measurements in a Scanning Proton Therapy Room: Can Parents Remain Near Their Children During Treatment?

**DOI:** 10.3389/fonc.2022.903706

**Published:** 2022-07-14

**Authors:** Vladimir Mares, Jad Farah, Marijke De Saint-Hubert, Szymon Domański, Carles Domingo, Martin Dommert, Magdalena Kłodowska, Katarzyna Krzempek, Michał Kuć, Immaculada Martínez-Rovira, Edyta Michaś, Natalia Mojżeszek, Łukasz Murawski, Ondrej Ploc, Maite Romero-Expósito, Marco Tisi, François Trompier, Olivier Van Hoey, Laurent Van Ryckeghem, Marek Wielunski, Roger M. Harrison, Liliana Stolarczyk, Pawel Olko

**Affiliations:** ^1^ Helmholtz Zentrum München, Institute of Radiation Medicine, Neuherberg, Germany; ^2^ Institut de Radioprotection et de Sûreté Nucléaire (IRSN), PSE-Santé, Fontenay-aux-Roses, France; ^3^ Belgian Nuclear Research Center, (SCK CEN), Institute for Environment, Health and Safety (EHS), Mol, Belgium; ^4^ National Centre for Nuclear Research, Radiological Metrology and Biomedical Physics Division, Otwock-Świerk, Poland; ^5^ Departament de Física, Universitat Autònoma de Barcelona, Bellaterra, Spain; ^6^ Cambridge University Hospital National Health Service (NHS) Trust, Medical Physics, Cambridge, United Kingdom; ^7^ Institute of Nuclear Physics, Polish Academy of Sciences, (IFJ PAN), Krakow, Poland; ^8^ Department of Radiation Dosimetry, Nuclear Physics Institute of the Czech Academy of Sciences (CAS), Prague, Czechia; ^9^ The Skandion Clinic, Uppsala, Sweden; ^10^ Faculty of Medical Sciences, University of Newcastle upon Tyne, Newcastle upon Tyne, United Kingdom; ^11^ Danish Centre for Particle Therapy, Aarhus University Hospital (AUH), Aarhus, Denmark

**Keywords:** scanning proton therapy, anthropomorphic pediatric phantom, secondary neutrons, active neutron monitors, ambient dose equivalent, clinical conditions

## Abstract

**Purpose:**

This study aims to characterize the neutron radiation field inside a scanning proton therapy treatment room including the impact of different pediatric patient sizes.

**Materials and Methods:**

Working Group 9 of the European Radiation Dosimetry Group (EURADOS) has performed a comprehensive measurement campaign to measure neutron ambient dose equivalent, H*(10), at eight different positions around 1-, 5-, and 10-year-old pediatric anthropomorphic phantoms irradiated with a simulated brain tumor treatment. Several active detector systems were used.

**Results:**

The neutron dose mapping within the gantry room showed that H*(10) values significantly decreased with distance and angular deviation with respect to the beam axis. A maximum value of about 19.5 µSv/Gy was measured along the beam axis at 1 m from the isocenter for a 10-year-old pediatric phantom at 270° gantry angle. A minimum value of 0.1 µSv/Gy was measured at a distance of 2.25 m perpendicular to the beam axis for a 1-year-old pediatric phantom at 140° gantry angle.

The H*(10) dependence on the size of the pediatric patient was observed. At 270° gantry position, the measured neutron H*(10) values for the 10-year-old pediatric phantom were up to 20% higher than those measured for the 5-year-old and up to 410% higher than for the 1-year-old phantom, respectively.

**Conclusions:**

Using active neutron detectors, secondary neutron mapping was performed to characterize the neutron field generated during proton therapy of pediatric patients. It is shown that the neutron ambient dose equivalent H*(10) significantly decreases with distance and angle with respect to the beam axis. It is reported that the total neutron exposure of a person staying at a position perpendicular to the beam axis at a distance greater than 2 m from the isocenter remains well below the dose limit of 1 mSv per year for the general public (recommended by the International Commission on Radiological Protection) during the entire treatment course with a target dose of up to 60 Gy. This comprehensive analysis is key for general neutron shielding issues, for example, the safe operation of anesthetic equipment. However, it also enables the evaluation of whether it is safe for parents to remain near their children during treatment to bring them comfort. Currently, radiation protection protocols prohibit the occupancy of the treatment room during beam delivery.

## Introduction

In recent years, tremendous technical progress has enabled proton therapy facilities to become more compact and cost-effective. Their clinical applications have expanded beyond brain and eye tumors, and this has drastically increased the number of patients receiving such treatment worldwide (1). Stray neutron radiation inherent to proton therapy remains, however, a topic of concern for the protection of both patients, especially pediatric patients (higher sensitivity and longer life expectancy), and healthcare professionals (shielding design) (2–6).

Dose limits recommended by the International Commission on Radiological Protection (7) are specified in terms of the protection quantity effective dose, E. These limits ensure that individuals are not exposed to unnecessarily high doses and so are a fundamental component of radiation protection in most countries. This protection quantity—effective dose—is not measurable. This means that an operational quantity—ambient dose equivalent, H*(10)—is used instead as a conservative estimate of effective dose, E. Such an approach also applies for estimating stray radiation exposures in radiotherapy including proton and ion therapy. The limits are split into two groups, public and occupationally exposed workers. Within Europe for the public, the effective dose limit is 1 mSv/year (higher values are allowed in a single year if the average over 5 years is not above 1 mSv/year), while for the occupationally exposed workers, it is 20 mSv/year, averaged over defined periods of 5 years with no single year exceeding 50 mSv. In the U.S., the Nuclear Regulatory Commission (NRC) requires to limit the occupational exposure to 50 mSv per year. Dose limits do not apply to medical exposures; however, the concept of radiation protection is still relevant.

Many authors have used Monte Carlo (MC) simulations and/or experimental tools to determine and model stray neutrons in scattering and scanning proton therapy (8–12). In a continuous effort to assess neutron exposure in proton therapy, Working Group 9 of the European Radiation Dosimetry Group (EURADOS WG9—Radiation dosimetry in radiotherapy) has performed a comprehensive intercomparison exercise to estimate neutron spectra and ambient dose equivalent around children treated using a spot scanning technique. At first, the work focused on determining neutron variability around a water phantom for a 10 × 10 × 10 cm^3^ target (13). Next, neutron variability with beam parameters (energy, field size, modulation width) was measured and a simplistic parametric model describing neutron doses around the phantom was suggested (14). For these measurements, extended-range Bonner sphere spectrometry systems, neutron rem counters, and tissue-equivalent proportional counters were used and benchmarked to help in selecting the optimal detector for proton therapy neutron spectra (15). In addition to environmental measurements, EURADOS WG9 also measured neutron doses in both water and anthropomorphic phantoms, using bubble, etched track, thermoluminescent, and radiophotoluminescent detectors (16–20).

Knezevic and colleagues have measured secondary neutron dose equivalent in pediatric phantoms during a simulated brain tumor treatment in the pencil beam scanning (PBS) proton facility at the Cyclotron Centre Bronowice, IFJ PAN Kraków (21). They observed a slightly higher neutron dose in a 10-year-old phantom compared to a 5-year-old phantom in all organs at distances from 20 cm to 30 cm from the isocenter. Nevertheless, the ambient dose equivalent dependence on patient size measured around the pediatric phantoms in the treatment room has not been yet systematically studied.

In this work, a brain tumor treatment, without a range shifter, was simulated using a set of pediatric anthropomorphic phantoms representing a 1-, 5-, and 10-year-old pediatric patient. Two different beam angles were considered to achieve clinically acceptable tumor coverage while optimizing the sparing of healthy organs at risks. Neutron stray radiation measurements were hence performed around the phantoms at eight different locations using the same set of active neutron monitors as previously benchmarked.

## Materials and Methods

### Pediatric Anthropomorphic Phantoms

For this study, three CIRS ATOM^®^ anthropomorphic phantoms representing 1-, 5-, and 10-year-old children were used (CIRS—Computerized Imaging Reference Systems, Inc., Norfolk, VA, USA). CIRS ATOM^®^ phantoms comprise 25-mm-thick sections with minimal interfaces between the slabs. The 1-year-old phantom is provided with arms and legs as a standard configuration, while arms and legs for 5-year and 10-year models can be fitted separately. The size and weight of each model is based on ICRP 23 (22), ICRU 48 (23), and available anatomical references (see **Table 1**). CIRS ATOM^®^ phantoms are constructed from materials simulating average soft tissue, average bone tissue, cartilage, spinal cord, spinal disks, lung, brain, and sinus. Simulated bone tissue for pediatric models matches age-related density.

**Table 1:** Anatomical references of CIRS ATOM^®^ 1-, 5-, and 10-year-old pediatric anthropomorphic phantoms used in this study, based on ICRP 23 (22), ICRU 48 (23) and available anatomical reference data.

Pediatric phantom	Height (cm)	Weight (kg)	Thorax dimension (cm × cm)
1 year	75	10.0	12 × 14
5 years	65*	13.1*	14 × 17
10 years	80*	21.5*	17 × 20

(^©^2015 Computerized Imaging Reference Systems, Inc.).*Without legs and arms.

### Proton Beam Specification, Irradiation Technique, and Irradiation Plans

**Figure 1:**
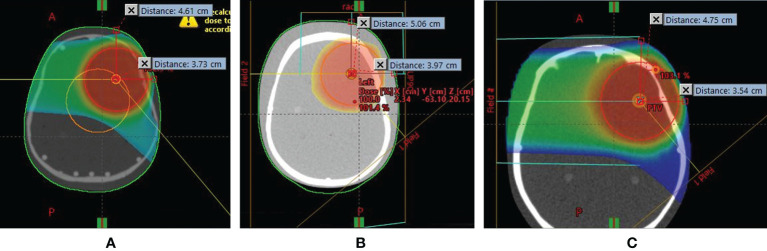
Position of intracranial tumor inside the left hemisphere of **(A)** 1-year-old, **(B)** 5-year-old, and **(C)** 10-year-old pediatric anthropomorphic phantoms.

The experiment was carried out at the Cyclotron Center Bronowice (CCB), which is a part of the Henryk Niewodniczański Institute of Nuclear Physics of Polish Academy of Sciences (IFJ PAN) in Kraków, Poland. The center is equipped with the Proteus C-235 cyclotron (IBA, Ion Beam Applications S.A., Belgium) able to accelerate protons for clinical use up to 226 MeV. Three treatment rooms have been available at CCB since 2016 for proton radiotherapy of cancer patients. These are two IBA 360° gantries with dedicated Pencil Beam Scanning (PBS) nozzles and a horizontal 70-MeV eye line. Computed tomography (CT) scans of CIRS phantoms representing 1-, 5-, and 10-year-old children were performed with the Siemens Somatom Definition AS Open scanner with a slice thickness of 0.2 cm, and then used in an Eclipse 13.6 Treatment Planning System (Varian Medical Systems) for preparation of irradiation plans calculated with the Proton Convolution Superposition (PCS) algorithm. The distance of the gantry nozzle to the isocenter inside the tumor was 46 cm. The spot size varied depending on the beam energy and depth inside the phantom. In the air at a distance of 46 cm from the gantry nozzle, 100-MeV and 140-MeV proton beams led to spot sizes of 5.3 mm and 4.4 mm, respectively. Two fields (proton beam directions) were applied to uniformly irradiate the 6-cm-diameter spherical target (5 cm tumor diameter plus 1 cm margin) situated inside the left hemisphere of the head (intracranial tumor) (see **Figure 1**). The isocenter was located in the middle of slice #3 of the CIRS phantom. The detailed position of the isocenter is shown in **Figure 1**.

For each pediatric phantom, a specific irradiation plan was prepared. In the case of the 5-year-old phantom for the first field, 2,231 individual proton beams (spots) in 27 layers were deposited at a gantry position of 270° with a maximal and minimal energy of 137.7 MeV and 84.0 MeV, respectively. The second field was irradiated at a gantry angle of 140° using 2,168 spots in 30 layers, with a maximal and minimal energy of 127.5 MeV and 71.6 MeV, respectively. Proton beam specifications for all irradiation plans are given in **Table 2**. A high proton dose was required to create neutrons measurable with acceptable precision. Thus, a total physical dose of ~100 Gy was delivered to the target volume for the 5-year and 10-year phantoms and ~40 Gy for the 1-year phantom. The dose was delivered from two beam directions with 60% of the dose with gantry position at 270° and 40% of the dose with gantry position at 140°. For reference dosimetry, a Semiflex-type ionization chamber (PTW 31010, Freiburg, Germany) with a Unidos Webline electrometer (PTW-Freiburg, Germany) was used together with an RW3 slab phantom.

**Table 2:** Proton beam specification.

Pediatric phantom	1 year		5 years		10 years	
Proton beam direction	140°	270°	140°	270°	140°	270°
E_min_ (MeV)	71.9	76.8	71.6	84.0	70.4	99.28
E_max_ (MeV)	124.7	128.8	127.5	137.7	128.1	144.6
R80 (cm)	9.54	10.64	10.26	11.92	10.11	13.27

### Experimental Setup Within the CCB Kraków Gantry Room

**Figure 2:**
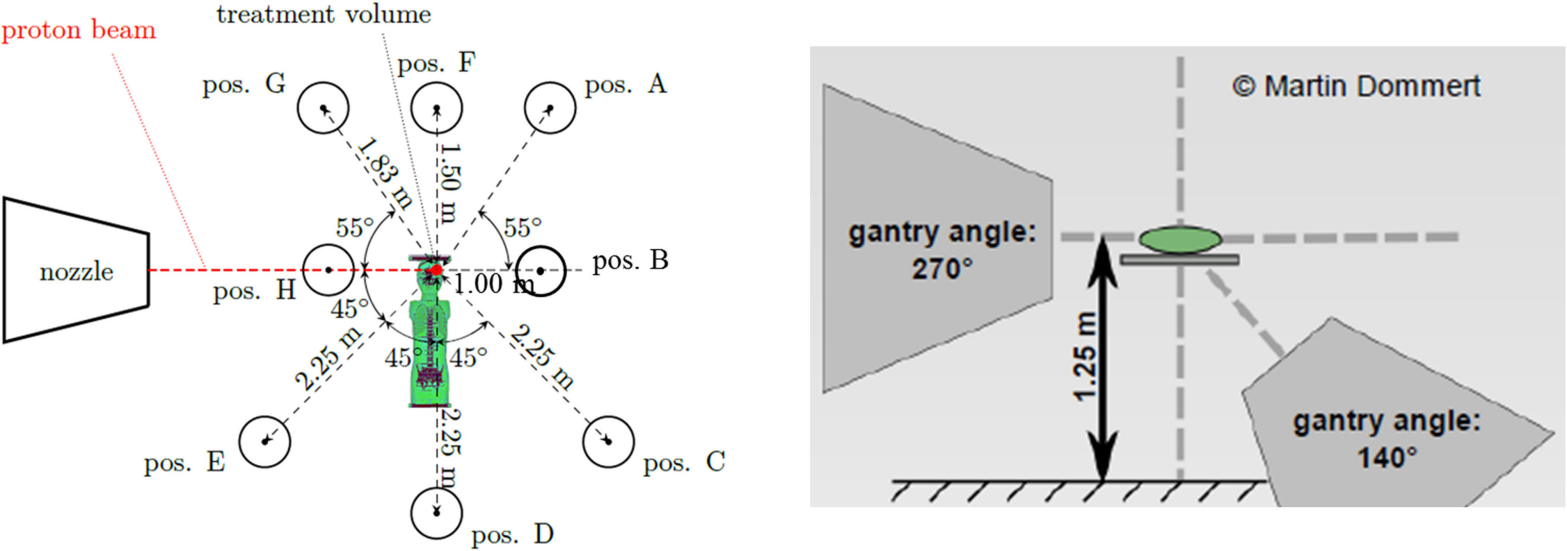
Schematic view of measurement positions around the 10-year-old pediatric phantom (left), and two gantry positions (right) (24).

**Figure 3:**
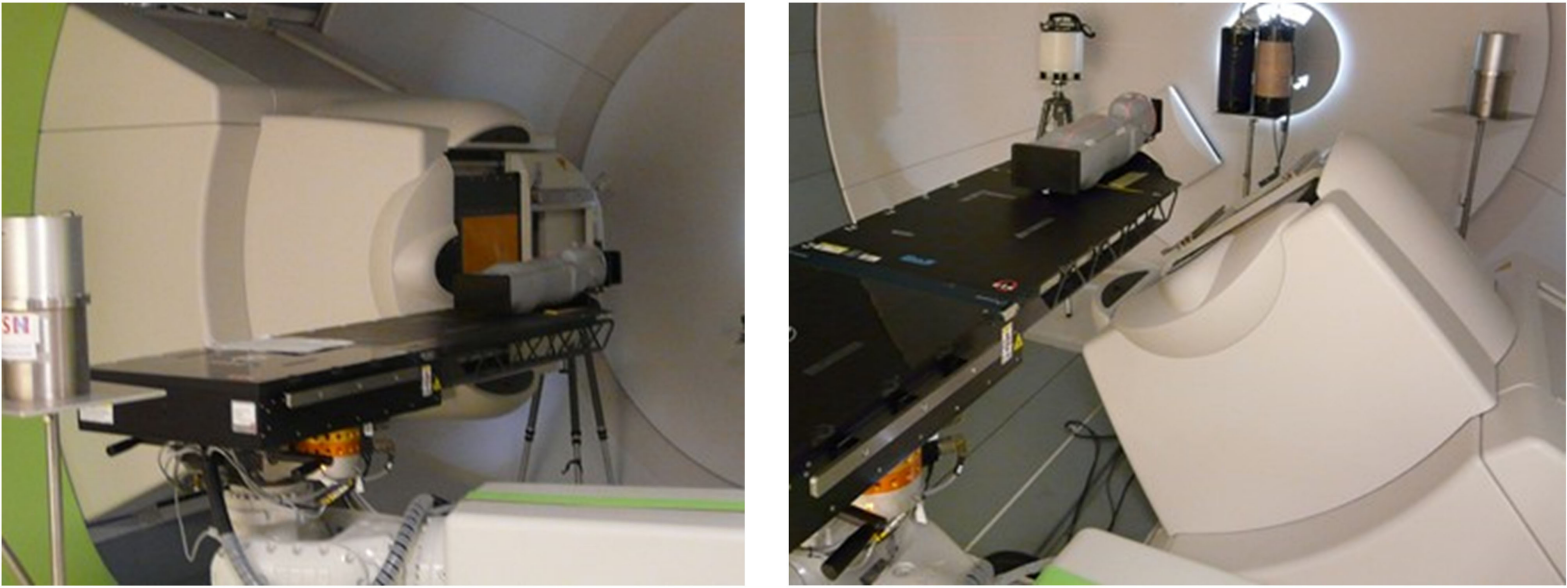
Picture of the experimental setup within the CCB gantry room with treatment nozzle at 270° (left) and 140° (right) Photo: V. Mares.

The experiment was carried out at CCB, focused on the creation of stray neutrons in conditions close to realistic treatment scenarios. Pediatric phantoms were placed on the therapeutic table perpendicular to the beam axis at an isocenter height of 1.25 m above the floor. The treatment was simulated using two fields (i.e., two gantry positions) at 140° and 270° angles (see **Figures 2**, **3**).

Neutron dose rates were mapped in the treatment room using different active neutron monitors. The ambient dose equivalents, H*(10), were measured at several distances and angular positions with respect to the beam axis around the 1-, 5-, and 10-year-old phantoms (see **Figure 2**), namely, along the beam direction (positions B and H), around the head (positions A, F, and G), and around the body (positions C, D, and E). The distances to the isocenter and angles with respect to the beam axis for all measurement positions are given in **Table 3**.

**Table 3:** Eight measurement positions around the 1-, 5-, and 10-year-old pediatric phantoms.

Position	Angle with respect to beam axis (°)	Distance to isocenter (m)
A	305	1.83
B	0	1.00
C	45	2.25
D	90	2.25
E	135	2.25
F	270	1.50
G	235	1.83
H	180	1.00

### Instruments

Neutron ambient dose equivalent, H*(10), around the pediatric phantoms was measured using several active neutron monitors: Hawk TEPC environmental monitors (Far West Technology, Inc.) from the Polish Institute of Nuclear Physics (IFJ), the French Institute for Radiological Protection and Nuclear Safety (IRSN), and the Czech Nuclear Physics Institute (NPI), and a TEPC chamber (Far West Technology, Inc.) from the Belgian Nuclear Research Centre (SCK CEN), as supplementary for the recombination chamber REM-2 type (POLON Bydgoszcz) with the GW2 ionization chamber from the National Centre for Nuclear Research (NCBJ). Various neutron rem counters were also used including the Berthold LB 6411 from Universitat Autonoma de Barcelona (UAB), Skandion Clinic, and the Belgian Nuclear Research Centre (SCK CEN), the Thermo Scientific™ WENDI-II from IFJ and IRSN, a conventional NM2B-458, and an extended-range NM2B-495Pb (NE Technology Ltd.) from Helmholtz Zentrum München (HMGU). Additionally, the Thermo Scientific™ RadEye™ NL from IRSN was applied. Further details are given in the Appendix.

**Figure 4:**
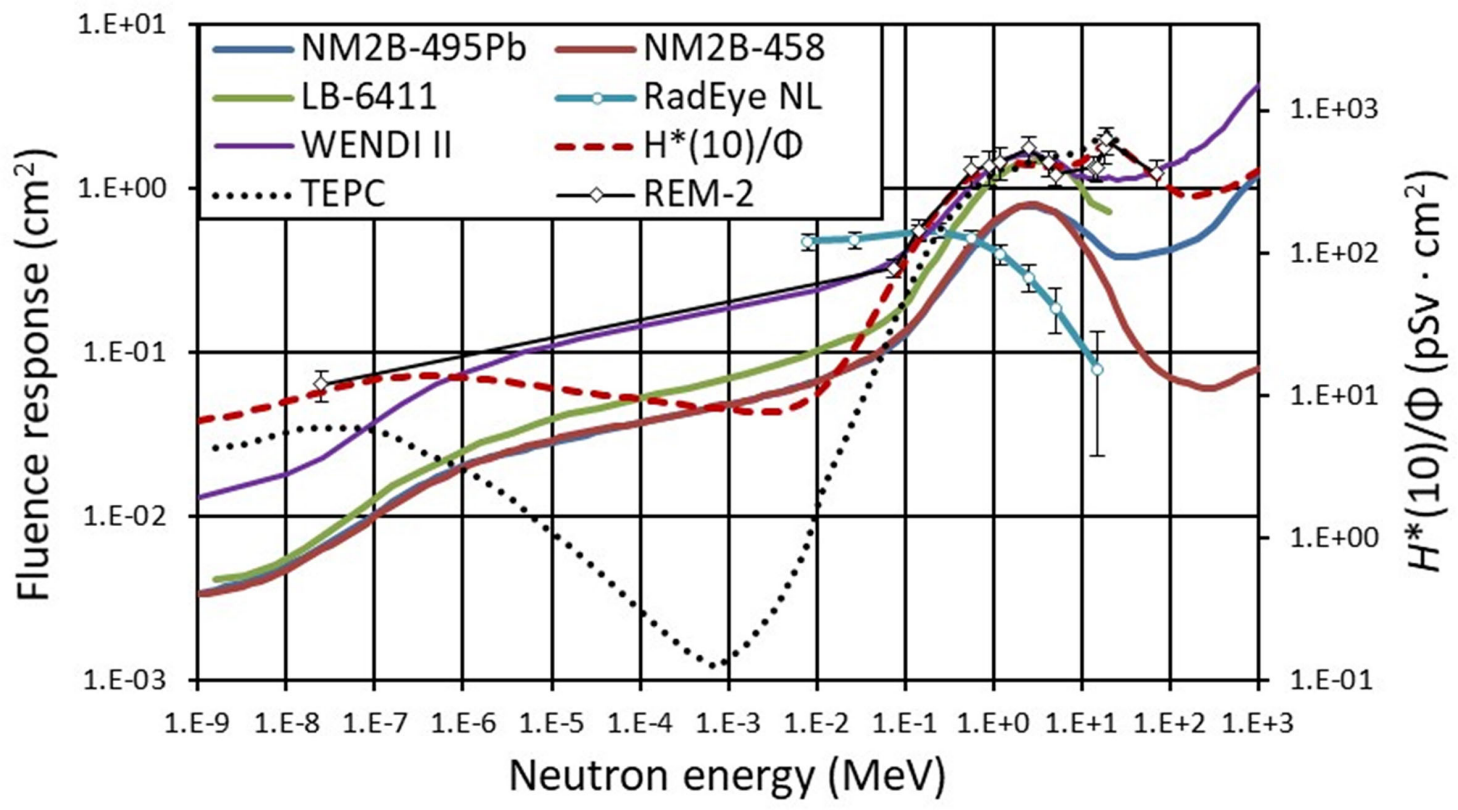
Neutron fluence response functions of rem counters NM2B-495Pb and NM2B-458 (25), Berthold LB-6411 (26), Wendi II (27), RadEye NL (28), TEPC (29), and REM-2 (30). Dashed line (red) represents H*(10) conversion coefficients for neutrons radiation versus neutron energy following ICRP74 recommendation (31) extended to high-energy neutrons according to Pelliccioni (32). The lines connect the points as a guide to the eye.

The most important neutron monitor criterion for the successful measurements in neutron fields with a wide energy range, typically from thermal up to several hundred MeV, is its fluence response as a function of neutron energy. In the most favorable case, it should follow the shape of the H*(10)/Φ energy dependence. In **Figure 4**, the neutron fluence response functions of the monitors used are plotted together with the H*(10)/Φ fluence-to-dose conversion coefficients as recommended by the International Commission on Radiological Protection 74 (31) and extended to high energies with data from Pelliccioni (32). According to the fluence response functions shown in **Figure 4**, it could be concluded that conventional neutron rem counters such as Berthold LB 6411 and NM2B-458 are not well suited for high-energy neutron fields as encountered in a proton therapy treatment room. Since high-energy neutrons contribute significantly to H*(10), conventional neutron rem counters (LB-6411 and NM2B-458) with decreased response to neutrons above 10 MeV and calibrated in Am-Be or Cf-252 fields considerably underestimate neutron H*(10) (13). The drop in the response of the RadEye™ NL pager for neutrons above about 1 MeV is also evident in **Figure 4**.

### Spectral Index

The same shape of the fluence response function of NM2B-495Pb and NM2B-458 rem counters from thermal neutrons up to about 10 MeV (see **Figure 4**) provides the unique possibility to swiftly estimate the contribution of high-energy neutrons to the total H*(10) without time-consuming neutron spectrometry. The so-called spectral index (SI) is here defined as a ratio of H*(10) measured with a high-energy extended rem counter NM2B-495Pb and conventional NM2B-458. In situations where neutron spectrometry could not be carried out because of time constraints for example, the SI value can provide a reliable first guess estimation of the spectrum shape and the corresponding contribution of high-energy neutrons to ambient dose equivalent, H*(10).

**Figure 5:**
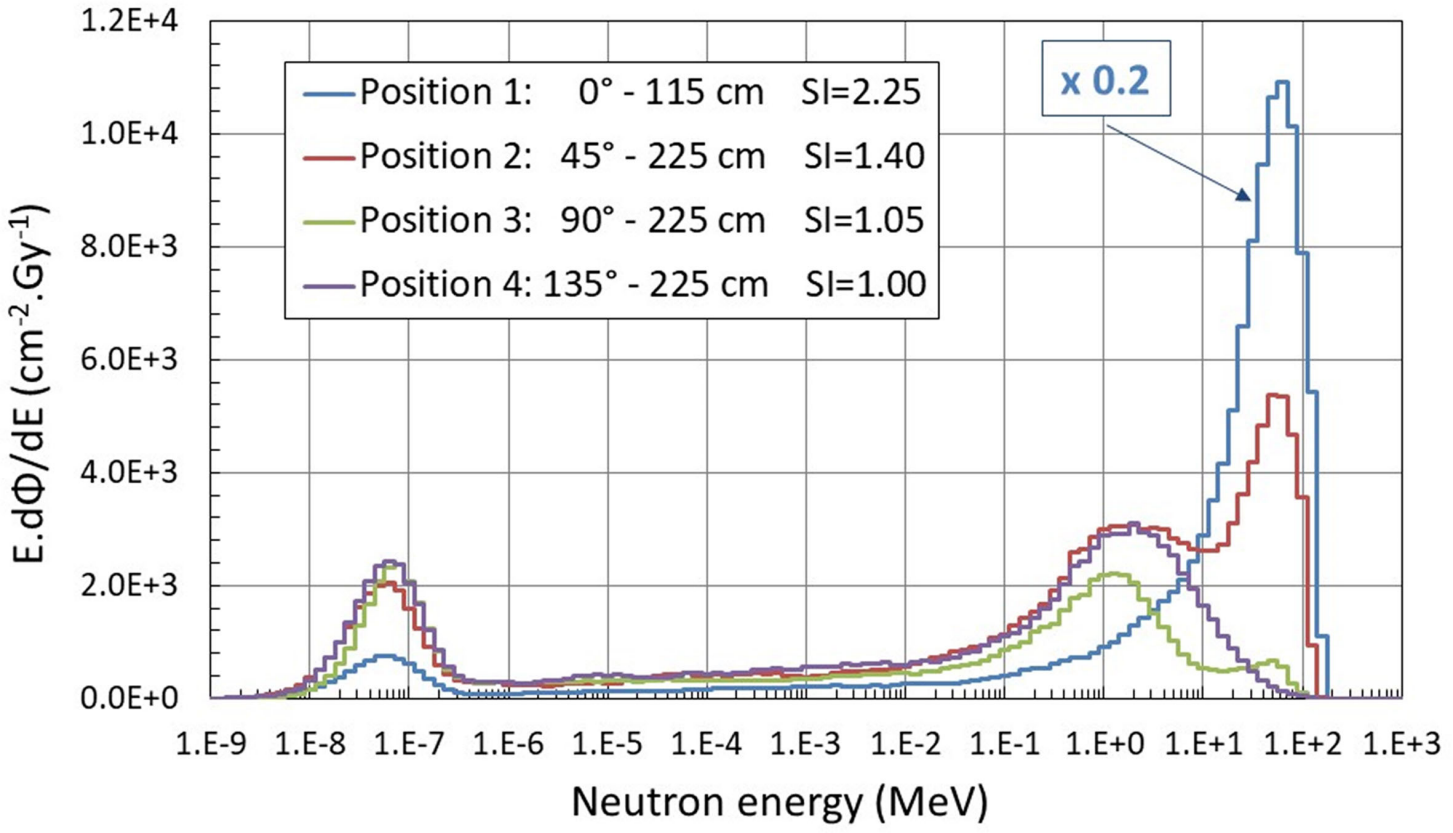
Neutron spectra measured around the water phantom in Trento PTC using the extended-range Bonner sphere spectrometer (13). Additionally, spectral index (SI) values calculated as ratio of H*(10) measured with a high-energy extended rem counter NM2B-495Pb and a conventional NM2B-458 are indicated.

Working group WG9 of EURADOS reported the results of a measurement campaign in the Trento proton therapy center (PTC) (13) where secondary neutron spectra were generated by a scanning proton beam targeting a cuboidal water tank phantom with dimensions of 30 × 30 × 60 cm^3^. Neutron spectra were recorded by extended-range Bonner sphere spectrometer (ERBSS) systems, and H*(10) values were assessed by the same NM2B rem counters, as used in this study, at four positions around the phantom (0°, 45°, 90°, and 135°). The Trento study showed that high-energy neutrons (>20 MeV) largely dominate the measured spectra along the beam axis (up to 60%) and drop with respect to the direction of the incident beam reaching 25% at 45°, 5% at 90°, and only 2% at 135° (see **Figure 5**). The spectral index in the Trento PTC was estimated to be equal to 2.25 along the beam axis (i.e., at 0°), 1.4 at 45°, 1.05 at 90°, and 1.0 at 135°.

Experimental conditions in the Trento and Kraków PTC are very similar and only differ by the beam size (10 × 10 cm^2^ square field in Trento versus 6 cm diameter in Kraków) and maximum proton energy (172 MeV in Trento versus 144 MeV in Kraków). Positions 1, 2, 3, and 4 in Farah et al. (13) correspond to positions B, C, D, and E in this study for gantry position at 270°.

## Results

### Spectral Index Values

**Figure 6:**
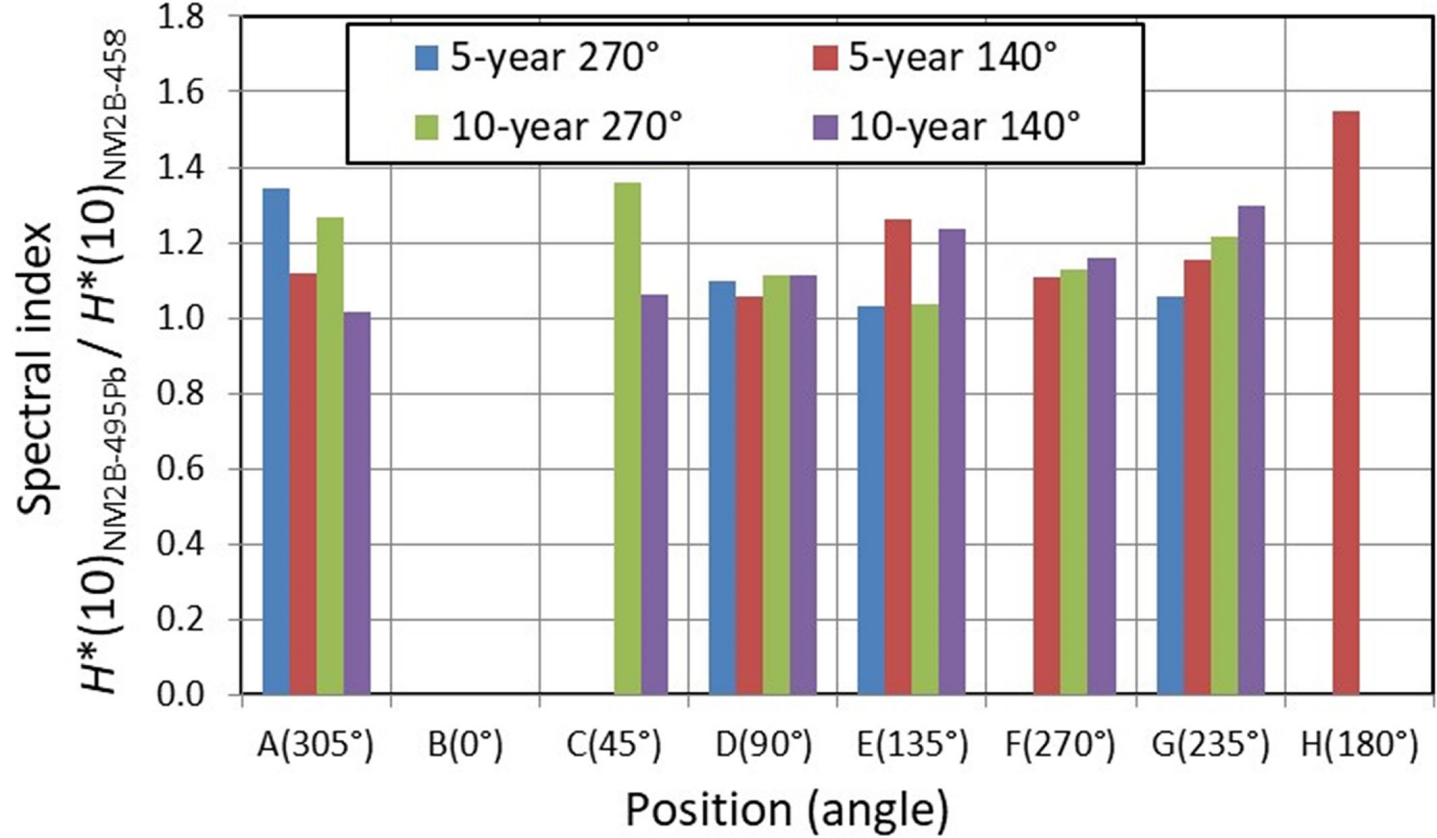
Neutron spectral index expressed as a ratio of H*(10) values measured with NM2B-495Pb and NM2B-458 rem counters at different positions (and different angles with respect to the beam direction) around 5- and 10-year-old pediatric phantoms.

The SI values assessed during the experiment in Kraków PTC are shown in **Figure 6**. The highest value of 1.55 was estimated for position H at the beam direction for the 5-year-old child in a gantry position of 140°. At positionto 1.36, while C at 45° (with 10-year-old child), the SI value was equal to 1.36, while at positions at larger angles with respect to beam direction, the SI values drop down to 1.10 (90°, position D, 5-year-old child) and 1.03 (135°, position E, 5-year-old child), respectively. It is noted that spectral index data for the 10-year-old child at position H and for the 5- and 10-year-old child at position B were not available, as well as all the data for the 1-year-old child.

The knowledge of the SI values has enabled data cleansing in the following way: at positions where SI was greater than or equal to 1.05, the H*(10) values measured with conventional detectors (i.e., applicable for neutrons below 10 MeV) were omitted from the dataset because of their underestimated values.

### Neutron Ambient Dose Equivalent Measured at Different Positions

**Figure 7:**
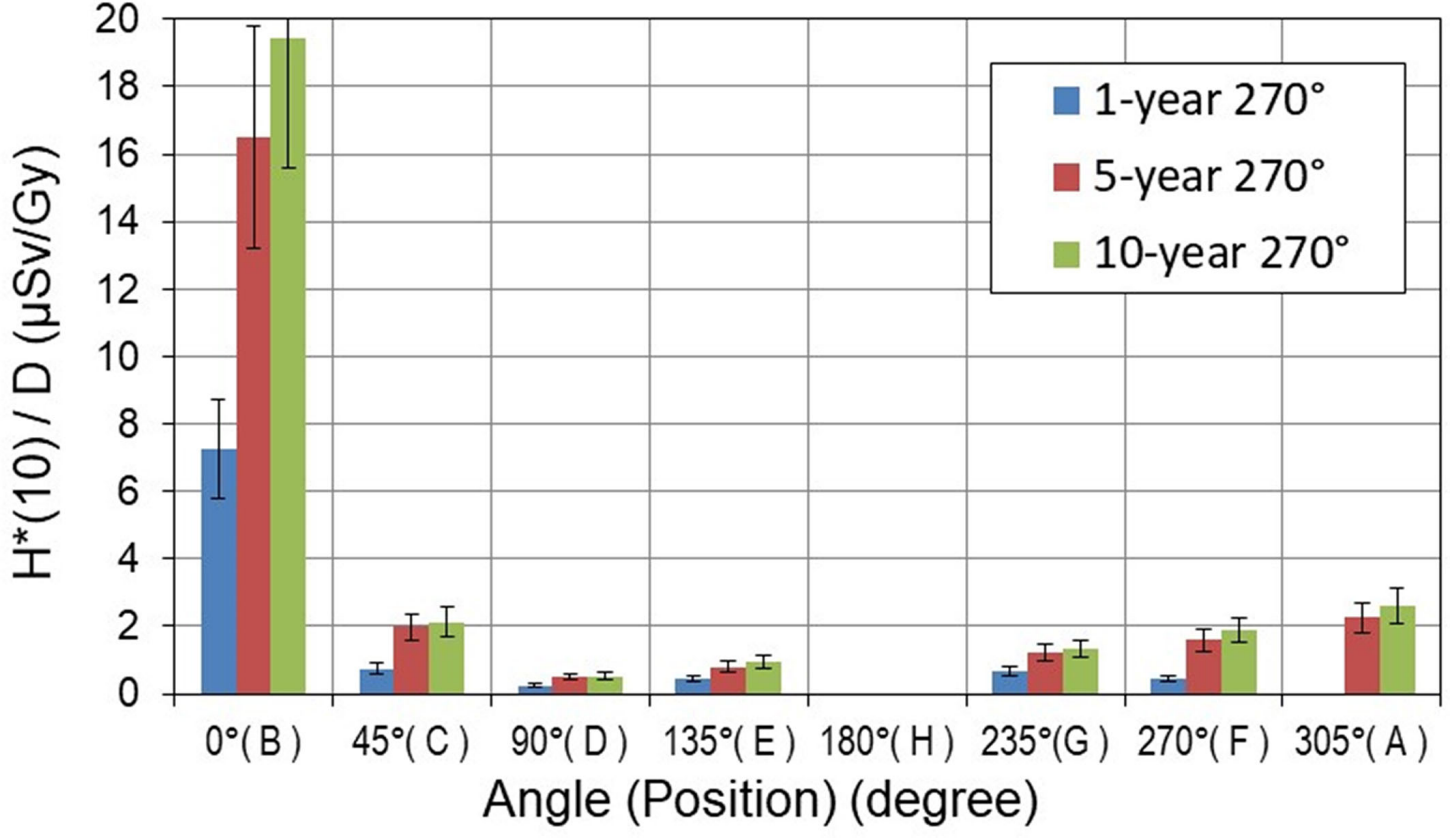
Neutron ambient dose equivalent H*(10) per treatment Gy [μSv/Gy] measured around pediatric phantoms at 270° gantry position. The error bars represent the standard deviation.

**Figure 8:**
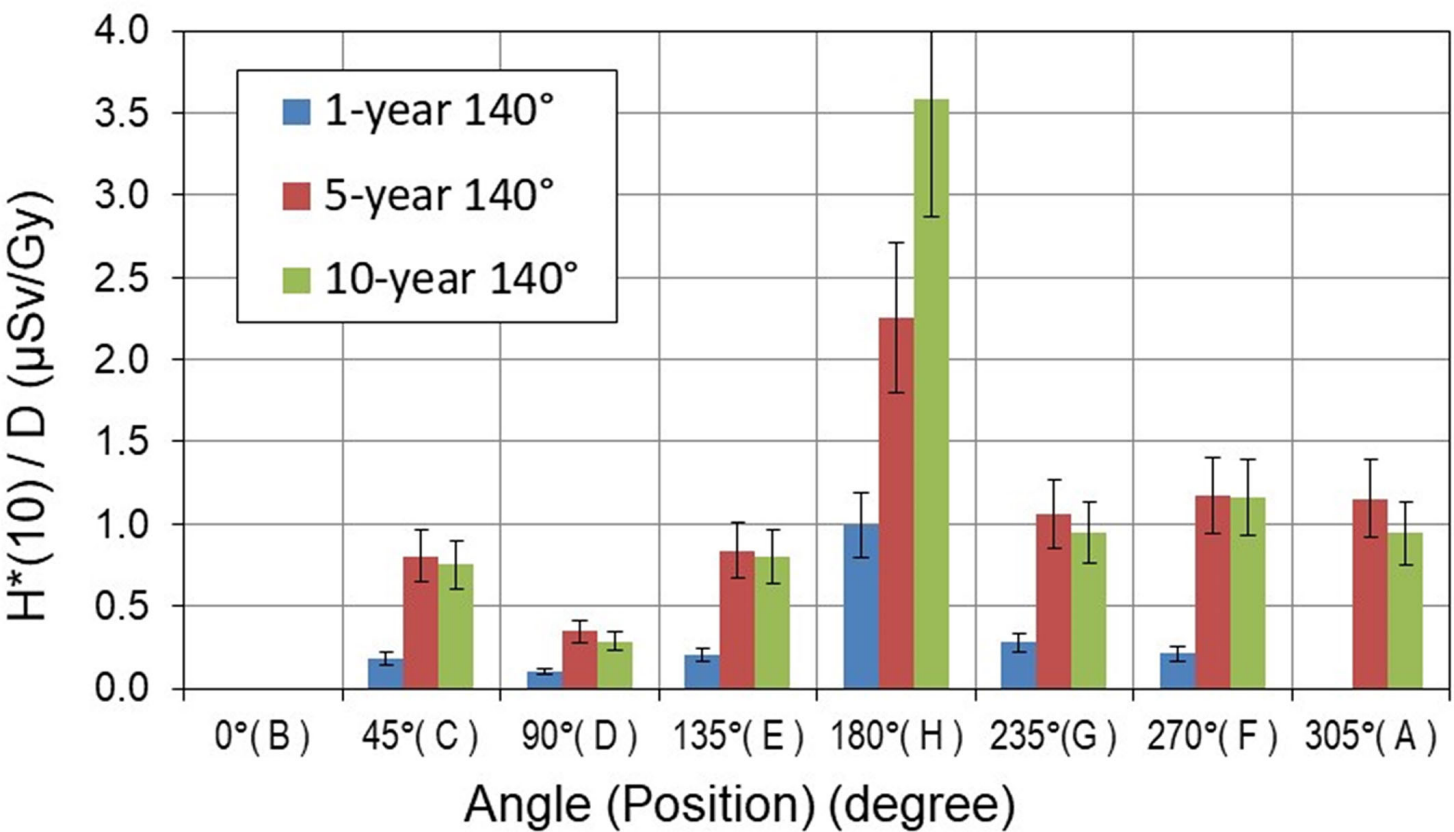
Neutron ambient dose equivalent H*(10) per treatment Gy [μSv/Gy] measured around pediatric phantoms at 140° gantry position. The error bars represent the standard deviation.

The aforementioned active detectors measured neutron H*(10) at each position under the same experimental conditions. It should be noted that at positions where SI ≥ 1.05, the H*(10) values measured with conventional rem counters (LB-6411 and NM2B-458) and RadEye™NL pager were omitted by data cleansing. For each specific position, the average value of all measured H*(10) was calculated and is shown in **Figure 7** per treatment Gy for the gantry position of 270° and in **Figure 8** for the gantry position of 140° for three pediatric phantoms used.

The H*(10) values show a significant decrease with both distance and angular position with respect to the beam axis. The highest neutron H*(10) value of 19.5 µSv/Gy was measured along the beam axis at a distance of 1.0 m from the isocenter (position B) for the 10-year-old child at 270° gantry position. The minimum H*(10) value of 0.1 µSv/Gy was measured at a distance of 2.25 m perpendicular to the beam axis (position D) for the 1-year-old child and for a 140° gantry angle.

The differences between H*(10) values in **Figures 7**, **8** clearly show the influence of specific proton beam parameters such as beam direction, maximal proton energy, and range of protons (see **Table 2**). It should be noted that the energy of protons determines both the maximum energy of produced neutrons and also the range of protons. However, the range of each spot also depends on the density of the material on the proton path. To characterize the range of the proton field, R80 of the depth dose profile along the main axis of the beam was measured in the treatment planning system (TPS). For example, in the 1-year-old phantom, R80 for the field with energies 77–129 MeV is about 10.6 cm, while for the 10-year-old phantom, R80 for the field with energies 99–145 MeV increases to about 13.3 cm, which means that for a higher proton energy, a higher amount of secondary neutrons is generated along the longer proton path and a higher H*(10) is observed.

At 270° gantry position, measured H*(10) values are below 2.1 µSv/Gy at all positions except along the beam direction (position B). In contrast, at 140° gantry position, H*(10) values are below 1.1 µSv/Gy except at position H (i.e., in beam direction). It should be noted that measurements at positions H and B were not possible at gantry angles 270° and 140°, respectively, because of spatial limitations (see **Figure 3**).

### Total Neutron Ambient Dose Equivalent

**Figure 9:**
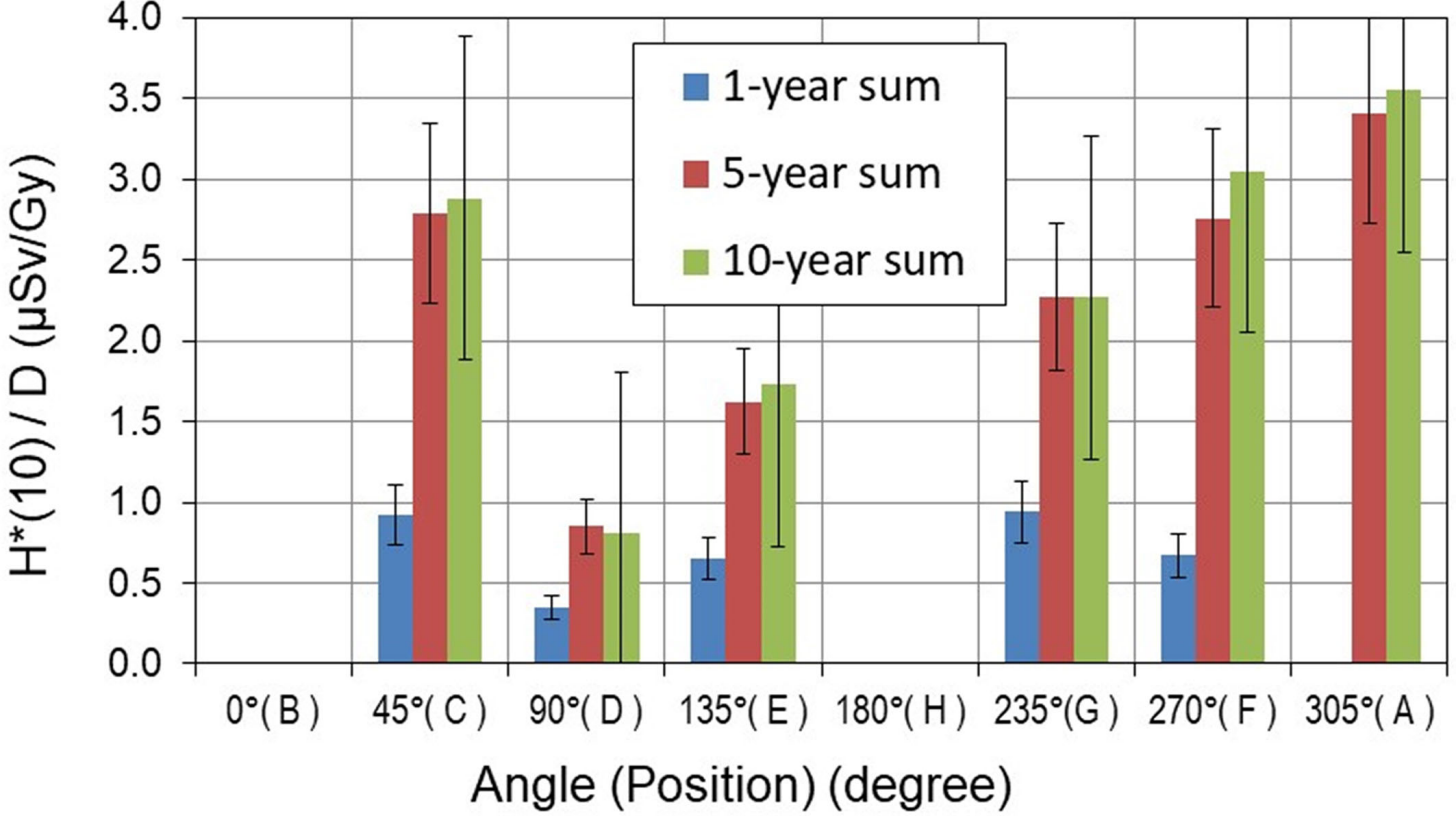
Total neutron ambient dose equivalent H*(10) per treatment Gy [μSv/Gy] measured around pediatric phantoms. The error bars represent the standard deviation.

The sum of H*(10) values for two proton beam directions at 140° and 270° gantry positions is shown in **Figure 9**. The H*(10) sum value at positions B and H cannot be shown as, because of spatial limitations (see **Figure 3**), it was not possible to measure H*(10) at both positions for both beam directions. It could be seen that during treatment with two fields, the total H*(10) did not exceed 1.0 µSv/Gy at positions perpendicular to the beam axis at a distance of 2.25 m.

### Impact of the Pediatric Patient Size

The H*(10) dependence on the size of the pediatric patient could be observed in **Figures 7**–**9**. At 270° gantry position, H*(10) values for the 10-year-old child were up to 20% and up to 410% higher than those measured for the 5- and 1-year-old child, respectively. At 140°, the patient size dependence of H*(10) for the 10- and 5-year-old child was markedly less prominent except at the position along the beam axis at 1-m distance from the isocenter (position H) where for the 10-year-old child increase of about 60% above H*(10) for the 5-year-old child was measured. The H*(10) values for the 10-year-old child were up to a factor of 5.5 higher than that for the 1-year-old child.

## Discussion

The measured ambient dose equivalent, H*(10), at positions along the beam axis, i.e., positions H and B, does not exceed 20 µSv/Gy, and drops significantly to about 1 µSv/Gy and 3 µSv/Gy for positions perpendicular to the beam axis (i.e., positions D and F), respectively. It means that the total neutron exposure of a person located at a position perpendicular to the beam axis at a distance greater than 2 m from the isocenter (e.g., at position D) does not exceed 60 µSv during the whole treatment course for a total target dose of 60 Gy (in 30 fractions). This dose remains well below the annual dose limit of 1 mSv for the general public (recommended by the International Commission on Radiological Protection). It should be noted that the H*(10) of 60 µSv is comparable with a calculated effective dose of 52 µSv received by passengers from galactic cosmic rays on a single flight from Munich to New York (~9 h flight duration) in the time period of solar minimum (33) using fluence-to-dose conversion coefficients as recommended in ICRP Publication 103 (7). In other words, for the investigated treatment plan, the unwanted exposure, due to a presence in the room 2 m from the isocenter during an entire spot scanning proton radiotherapy treatment (30 fractions), is by at least an order of magnitude lower than the annual dose limit for the public.

This comprehensive analysis of variability of H*(10) is of key importance for neutron shielding and, for example, for safe operation of anesthetic equipment. Moreover, it also enables the evaluation of whether it is safe for parents to remain near their children during treatment to bring them comfort, which could even avoid anesthesia during treatment and/or reduce movement during treatment.

Nevertheless, current work does not yet allow the generalization of such practices, as neutron H*(10) depends on treatment plan parameters such as size of the target, patient position, number of beams, beam incidence, and proton energies (14). Future work will be needed to extensively study the impact of such parameters in order to generalize findings to ensure appropriate shielding, the safe operation of anesthesia, and the safe presence of parents during treatment of their children.

Another limitation is that, even though the experiment was conducted under conditions close to realistic treatment scenarios, no range shifter was used, as during the experiment, the range shifter was not fully commissioned. Therefore, for one of the treatment fields, a range shifter could not be used, and the tumor, located in the left hemisphere of the brain, was irradiated from the right side (270° angle) instead of the choice for a left-sided beam orientation. As such, the proton energy was slightly increased to reach the appropriate depth in brain and the clinical translatability is challenged. Even though this can be considered a limitation of the study, we believe that the corresponding neutron H*(10) can be considered as a conservative estimation of the H*(10), as it is increasing with increasing proton energy (14), and general findings of the paper are consistent.

## Conclusions

The measurements performed to investigate the secondary neutron dose around 1-, 5-, and 10-year-old children in clinical PBS proton therapy showed that the size of the pediatric patient influences the magnitude of the neutron ambient dose equivalent at various positions in the treatment room. The clear dependence of H*(10) values on the size and age of the pediatric patient was observed mainly for the 270° proton beam direction (gantry position at 270°). In this case, H*(10) values for the 10-year-old child were up to 20% higher than those measured for the 5-year-old child and up to 290% higher than for the 1-year-old child.

This study also showed that the neutron ambient dose equivalent H*(10) decreases with distance from the isocenter and strongly depends on the position angle with respect to the beam axis. The highest H*(10) values were always measured along the beam axis, while the lowest H*(10) values were measured at positions located perpendicularly to the beam axis. The highest neutron ambient dose equivalent of about 19.5 µSv/Gy was measured at a distance of 1.0 m from the isocenter along the beam axis (i.e., at closest point during experiment) at a gantry position of 270° for the 10-year-old pediatric phantom. H*(10) values significantly decreased to 0.1 µSv/Gy at a distance of 2.25 m perpendicular to the beam axis for a 1-year-old pediatric phantom at a gantry position of 140°.

It was also demonstrated that during the whole treatment course with a target dose ≤ 60 Gy, the total neutron exposure of a person at a position perpendicular to the beam axis at a distance of 2.25 m remains well below the annual dose limit for the public. For the specific conditions of this study, it may be concluded that parents could remain 2 m away from their children to bring them comfort and possibly limit risks of patient motion during therapy, which could jeopardize treatment quality. Currently, the radiation protection protocols prohibit the occupancy of the treatment room during beam delivery. The very low doses demonstrated here suggest that for proton therapy under the conditions described in this paper, the procedures and practices could be re-assessed. However, further work is required before definitive guidance on parental occupancy of the treatment room could be given.

## Data Availability Statement

The raw data supporting the conclusions of this article will be made available by the authors, without undue reservation.

## Author Contributions

VM: participation in measurement campaign, data evaluation, experiment management, data analysis, drafting and editing manuscript. JF: participation in measurement campaign, data evaluation, experiment management, data analysis, drafting and editing manuscript. MS-H: participation in measurement campaign, data evaluation, data analysis, drafting and editing manuscript. SD: participation in measurement campaign, data evaluation, drafting and editing manuscript. CD: participation in measurement campaign. MD: participation in measurement campaign. MaK: participation in measurement campaign, data evaluation, drafting and editing manuscript. KK: data evaluation. MiK: data evaluation. IM-R, participation in measurement campaign, drafting and editing manuscript. EM: data evaluation. NM, participation in measurement campaign, irradiation plan, drafting manuscript. LM: participation in measurement campaign. OP: participation in measurement campaign, data evaluation. MR-E: participation in measurement campaign, data evaluation, drafting and editing manuscript. MT: participation in measurement campaign. FT: data evaluation, data analysis, drafting and editing manuscript. OH: participation in measurement campaign, data evaluation, data analysis, drafting and editing manuscript. LR: participation in measurement campaign. MW: participation in measurement campaign. RH: participation in measurement campaign, data analysis, drafting and editing manuscript. LS: participation in measurement campaign, irradiation plan, experiment management, data analysis, drafting and editing manuscript. PO: participation in measurement campaign, experiment management, data analysis, editing manuscript. All authors contributed to the article and approved the submitted version.

## Conflict of Interest

The authors declare that the research was conducted in the absence of any commercial or financial relationships that could be construed as a potential conflict of interest.

## Publisher’s Note

All claims expressed in this article are solely those of the authors and do not necessarily represent those of their affiliated organizations, or those of the publisher, the editors and the reviewers. Any product that may be evaluated in this article, or claim that may be made by its manufacturer, is not guaranteed or endorsed by the publisher.

## Funding

I.M.-R. acknowledges the financial support from the Spanish Ministry of Science, Innovation and Universities (fellowship RYC2018-024043-I). The work of Ondrej Ploc on the paper was funded by EU Operational Program Research, Development, and Education, call 02_15_003 in project CRREAT, number CZ.02.1.01/0.0/0.0/15_003/0000481. The study was partially supported by the EU Project POWR.03.02.00-00-I004/16 and the Horizon 2020 project INSPIRE Grant Agreement 730983.

## Appendix

**A. Instruments**

**A-1: NM2B-495Pb and NM2B-458 rem counter**

NM2B-458 and NM2B-495Pb (NE Technology Ltd., commercially available as an instrument pair NM500 and NM500X from Münchener Apparatebau für elektronische Geräte, GmbH) are cylindrical Andersson-Braun rem counters measuring neutron ambient dose equivalent, *H**(10). Both rem counters are based on cylindrical BF_3_ proportional counters of 3.1 cm outer diameter and 7.2 cm active length surrounded by an inner polyethylene moderator (1.7 cm thick), a 0.6-cm-thick boron-doped synthetic rubber absorber, and an outer polyethylene moderator (6.9 cm thick). In the case of the NM2B-495Pb model, a 1-cm-thick lead shell surrounding the boron rubber is added to extend the detection range to high-energy neutrons.

The fluence response functions from thermal to 10 GeV were calculated by means of different Monte Carlo codes, i.e., MCNP (34) for energy below 20 MeV, and LAHET (35), HADRON (36), and MCNPX (37) above 20 MeV. Details are described in Mares et al. (25). All calibrations were performed in HMGU using 185 GBq (5 Ci) ^241^Am-Be (α,n) neutron source with an average neutron energy of 4.4 MeV (38–40). Both rem counters were also calibrated in 100 and 300 MeV quasi-mono-energetic neutron fields at RCNP in Osaka, Japan (41) and at CERF (https://tis-div-rp-cerf.web.cern.ch/). The measurement uncertainties were estimated to be ±20% for the NM2B-495Pb extended-range rem counter, and ±30% for the NM2B-458 conventional one.

In the present experiment in CCB Kraków, pulse height spectra were registered to control the photon background, to correct for pile-ups, and to properly set the region of interest (ROI) to evaluate appropriate number of counts. Applying the calibration factors estimated in HMGU, the ROI counts can be converted to corresponding ambient dose equivalent, *H**(10).

**A-2: LB 6411 Berthold**

The LB 6411 probe (Berthold Technologies) consists of a polyethylene moderator sphere with a diameter of 25 cm and a cylindrical ^3^He proportional counter at its center. This monitor is designed to measure neutron ambient dose equivalent *H**(10) in the neutron energy range from thermal to 20 MeV. It is known to have a strongly decreasing sensitivity to neutrons above 20 MeV. The relative dose response function of the LB 6411 over the whole energy range was calculated with MCNP Monte Carlo code by Burgkhardt et al. (26). The calculated response was benchmarked with measurements in monoenergetic neutron reference fields.

The ^252^Cf neutron source has been used for calibration. The calibration factor of 0.353 nSv/count is used to display the data as *H**(10). The response to gamma radiation is about 10^−3^ counts per nSv. The overall measurement uncertainty of about 30% mainly include the uncertainties in detector calibration, dose delivery, detector positioning, and the energy response of the detectors.

**A-3: WENDI-II**

The WENDI-II (manufacturer: Thermo Scientific) is an extended-range rem counter built by Olsher et al. (42). It has an outer diameter of 22.86 cm and consists of a ^3^He proportional counter surrounded by a cylindrical polyethylene assembly with an inner tungsten shell. Compared to a conventional rem counter, such as, e.g., the Berthold LB 6411, the sensitivity to high energy neutrons (E > 20 MeV) is significantly higher thanks to inelastic (*n,xn*) reactions occurring in the tungsten layer.

The ^252^Cf calibration constant of the WENDI-II used for these measurements is 0.317 nSv/count. The relative dose response function, calculated as the absolute response function multiplied by this calibration constant and divided by the fluence-to-*H**(10) conversion coefficients is shown in De Smet et al. (27, 43). The overall measurement uncertainty mainly including the uncertainties in detector calibration and the energy response of the detectors was estimated to be of about 20%.

**A-4: “HAWK” Tissue-equivalent proportional counter monitor**

The HAWK environmental Monitoring System FW-AD type 1 is a tissue-equivalent proportional counter from Far West Technology Inc. (Goleta, California, USA), composed of a spherical chamber (127 mm diameter) with a wall from A-150 tissue-equivalent plastic (2 mm thick) and filled with pure propane gas at low pressure (about 9.33 hPa) simulating a 2-μm site size (44). The outer container is made of 6.35-mm-thick stainless steel. The dose equivalent is calculated from a spectrum of single energy deposition events and a radiation quality factor Q, determined by the Q(L) relation given in ICRP 60 (45), where L denotes the unrestricted linear energy transfer (LET) in the exposed material (7).

HAWK type 1 systems use two linear multichannel analyzers working in parallel with low and high gains. The low-gain analog-to-digital converter (ADC) measures LET spectra up to 1024 keV·μm^−1^ with 1 keV·μm^−1^ resolution. The high-gain channel uses an ADC measuring up to a lineal energy of 25.6 keV·μm^−1^ with a resolution of 0.1 keV·μm^−1^. The energy deposition of the low-LET and high-LET components and the associated quality factor are stored in an output file once per minute. The separation between the low-LET and the high-LET component is set at 10 keV·μm^−1^ according to the Q(L) relationship (7). Events encountering significant electronic noise below the so-called low energy threshold (0.3 keV·μm^−1^ for the HAWK used here) are not recorded. For data analysis, a simple coefficient (the average of correction factor determined for ^60^Co and ^137^Cs gamma-rays) was applied (46). No compensation of the counting loss due to dead time is included in the analysis software.

Correction factors, N_low_ and N_high_, to ambient dose equivalent for the low-LET and high-LET components of the dose equivalent are used. N_low_ was determined in photon radiation fields with ^60^Co and ^137^Cs sources. N_high_ was defined using the neutron reference sources of ^241^Am–Be or ^252^Cf neutron sources. The values of N_low_ are 1.11 ± 0.02 and 1.34 ± 0.03, and the values of N_high_ are 0.80 ± 0.09 and 0.84 ± 0.10 for IRSN and SL, respectively. Correction coefficients for neutrons were also evaluated for neutron energies between 0.5 and 19 MeV and were found similar to Am–Be or ^252^Cf neutron sources (47).

**A-5: Tissue-equivalent proportional counter TEPC**

The TEPC (Model LET-SW5, Far West Technology) used for these measurements is spherical, with an internal diameter of 12.55 cm and a 2-mm-thick shell of A-150 tissue-equivalent plastic. The TEPC was filled with propane tissue-equivalent gas at a pressure of 8.8 mbar to simulate a biological site size of 2 µm. The relative dose response function of this type of detector was calculated up to 20 MeV by Thomas (29). The ambient dose equivalent *H**(10) was calculated as explained in Farah et al. (46). *H**(10) value uncertainties are in the order of 15% when adding statistical (10%), alpha calibration (10%), extrapolation (2%), proton beam delivery (5%), and detector positioning (1%) uncertainties.

**A-6: RadEye NL**

The RadEye™NL pager (Thermo Scientific™) is a small, lightweight, and highly sensitive radiation detection device that incorporates a ^3^He counter filled at 2.5 bars to detect very low radiation levels of neutron radiation from any source. A polyethylene shell moderator, provided by Thermo Scientific™ to improve the detection sensitivity, was used for these measurements. This moderator has a non-standard geometry (parallelepiped shape), which cannot ensure an isotropic response and limit its use as a neutron rem counter.

The response function of this detector was previously (28 studied using various types of reference sources (^241^Am-Be, bare and heavy water moderated ^252^Cf) as well as with several monoenergetic neutron fields available at the AMANDE facility (48). Hence, the variation of the response with neutron energy was determined at 8 keV, 27 keV, 144 keV, 250 keV, 565 keV, 1.2 MeV, 2.5 MeV, 5 MeV, and 15 MeV. The fluence reference values in the monoenergetic fields are established with an IRSN long counter traceable to national standards. The response of the RadEye™ NL pager was found to be similar to leak design counters, overestimating the *H**(10) values at the lowest energies and underestimating it at the highest energies. In this particular calibration, the response of the detector was set to unity for the ^252^Cf source while a 30% under-response is noticed for the ^241^Am-Be. It should be noticed, however, that the main limitation of this counter is the non-isotropic moderator geometry that induces a large angular dependence depending on neutron energy; namely, a factor of ~2 under-response was observed at 144 keV when comparing a 0° front exposure of the moderator and a 90° side exposure of the moderator; this under-response drops to ~10% at 15 MeV.

**A-7: GW2 and REM-2 ionization chamber**

A research group from the National Centre for Nuclear Research, during the experiment, used unique gas detectors. The recombination chambers are ionization chambers designed in such a way that, at a certain range of gas pressure and dose rates, the initial recombination of ions dominates over the volume recombination, when the chamber operates at polarizing voltages below saturation. In the aspect of scientific research, they are excellent devices for comparing methods and results with popular, commercially available neutron detectors.

The REM-2 type detector is a large recombination chamber filled with a gas mixture consisting of 95% methane and 5% nitrogen up to a pressure of 10 atm. The gas volume is 1800 cm^3^. The detector is a cylinder with outer dimensions of about 30 cm height and 15 cm diameter. Inside the detector, there are 25 parallel-plate tissue-equivalent 3-mm-thick electrodes with 7 mm space between. The total mass of the detector is about 6.5 kg with an effective wall thickness of about 2 g/cm^2^. Because of the large volume, the detector is very sensitive (~2.63 × 10^6^ Gy/C for Air Kerma Rate in the ^137^Cs isotopic radiation field). The device is a radiation sensor that allows one to determine radiation quality using the microdosimetric relationship between the initial recombination efficiency and local ion density. It has been proved (49, 50) that the recombination index of the radiation quality Q_4_ parameter is a good approximation of the Q(L) relation given in ICRP 60 (45). The main advantage of the chamber is the high compliance of the ambient dose equivalent at a depth of 10 mm *H**(10) for ICRU spheres on a wide range neutron ﬁeld energy spectrum from thermal up to 20 MeV and even further (30).

The tissue-equivalent REM-2 type detector and the non-hydrogen GW2-type gamma detector used in the experiment were placed side by side. The combination of hydrogen-free and tissue-equivalent detectors allows, independently from determining the radiation quality factor, the separation of the gamma and neutron components of mixed radiation (51, 52).

The GW2-type detector is an ionization chamber with aluminum electrodes and is filled with CO_2_ up to 26 atm pressure. Dimensions and number of electrodes are similar to the REM-2-type detector. Due to its construction, GW2 is almost insensitive to neutron radiation. In this experiment, the chamber had been used as a monitor for the gamma radiation component of the radiation field. The sensitivity of the detector is about ~9.58 × 10^6^ Gy/C for Air Kerma Rate in the ^137^Cs isotopic radiation field.

Before the experiment, both detectors were calibrated with the ^137^Cs reference photon source and REM-2 was additionally checked against the radiation quality of the isotopic Am–Be neutron field. The ambient dose equivalent *H**(10) calculation was adopted following Tulik et al. (52). Determined uncertainties of *H**(10) values are in the order of 25%.
